# Nitric Oxide Signaling Exerts Bidirectional Effects on Plasticity Inductions in Amygdala

**DOI:** 10.1371/journal.pone.0074668

**Published:** 2013-09-25

**Authors:** Ryong-Moon Shin, Makoto Higuchi, Tetsuya Suhara

**Affiliations:** Molecular Imaging Center, National Institute of Radiological Sciences, Anagawa 4-9-1, Inage-ku, Chiba, Japan; The Research Center of Neurobiology-Neurophysiology of Marseille, France

## Abstract

It has been well known that long-term potentiation (LTP) of synaptic transmission in the lateral nucleus of the amygdala (LA) constitutes an essential cellular mechanism contributing to encoding of conditioned fear. Nitric oxide (NO), produced by activation of the postsynaptic N-methyl-D-aspartate receptors (NMDAR) in thalamic input to the LA, has been thought to promote LTP, contributing to the establishment of conditioned fear. However, it is not known whether and how NO, released from cortical input to the LA, plays the role on the plasticity induction and fear memory. Here we report that the diffusion of NO, released in response to activation of presynaptic NMDAR on cortical afferent fibers in the LA, could suppress heterosynaptically a form of presynaptic kainate receptor (KAR) dependent LTP (pre-LTP) in thalamic input, which was induced by low-frequency presynaptic stimuli without postsynaptic depolarization. We also confirmed that NO, produced by activation of postsynaptic NMDAR in thalamic input, can promote postsynaptic NMDAR-dependent LTP (post-LTP), which was induced by pairing protocol. These LTPs were occluded following fear conditioning, indicating that they could contribute to encoding of conditioned fear memory. However, their time courses are different; Post-LTP was more rapidly formed than pre-LTP in the course of fear conditioning. NO, produced by activation of presynaptic NMDAR in cortical input and postsynaptic NMDAR in thalamic input, may control conditioned fear by suppressing pre-LTP and promoting post-LTP, respectively, in thalamic input to the LA.

## Introduction

In central neural system, such as hippocampus, NO has been widely believed to be synthesized in the postsynaptic cell by an enzyme NO synthase (NOS), which is activated directly by calmodulin via NMDAR-mediated influx of Ca^2+^ during LTP induction process. Once generated, NO is thought to signal from postsynaptic site to presynaptic terminals homosynaptically as retrograde factor and modulate the probability of neurotransmitter release by activation of cyclic guanosine monophosphate (cGMP), leading to the modification of synaptic efficacy in neural circuits. Importantly, NO signaling has been implicated in a number of hippocampus-dependent learning and memory processes. However, recent reports have shown that NO, probably produced by presynaptic NMDAR activation, may play as “anterograde factor” an important role on postsynaptic cell in regulation of long-term depression in cerebellar parallel fiber-Purkinje synapse [1]. NO, diffusible gas, is also required for heterosynaptic spread of cerebellar [2] and hippocampal LTP [3]. Presynaptic NMDAR mediated NO signaling, localized in GABAergic terminals, has been reported to inhibit the machinery for the probability of GABA release in hippocampus [4], suggesting that NO might act in an auto-synaptic manner.

The amygdala, one of the structures in the limbic system, is critical for the perception and expression of fear, as demonstrated by a study using functional magnetic resonance imaging [5]. In experimental animals [6], conditioned fear, resulting from learning of association between a neutral stimulus, audible tone, and an aversive stimulus, foot-shock, has been known to be primarily formed in the LA. The acquisition of fear memory is mediated by LTP-like synaptic enhancements in auditory afferents, including both cortical and thalamic inputs to the LA [7,8]. Consistent with the role of NMDAR within the LA in fear conditioning [9], numerous studies have demonstrated a close link between conventional NMDA-dependent LTP and conditioned fear [10,11]. Although it has been reported that NO might play a role in the induction of conventional LTP in thalamic input and the acquisition of conditioned fear [12,13], the role of NO on synaptic plasticity in cortical input is still under debate [14,15].

Here we addressed this issue by investigating whether and how NO could be synthesized at cortico-LA synapse. In addition, we also explored how NO, produced at cortico-LA synapse, might control encoding of conditioned fear in the LA.

We found that NO could be released in response to activation of presynaptic NMDAR on terminals of cortical input. We also showed that the induction of previously described form of presynaptic KAR-dependent LTP (pre-LTP) in thalamic input to the LA [16] was occluded following fear conditioning. Interestingly NO, released at cortico-LA synapse, could suppress heterosynaptically pre-LTP in thalamic input, suggesting the possibility that NO in cortical input might control the amount of learned fear.

## Materials and Methods

The animals used here were maintained and handled in accordance with the National Research Council *Guide for the care and Use of laboratory animals* and our institutional guidelines. Protocols for the present animal experiments were approved by the Animal Ethics Committee of the National Institute of Radiological Sciences.

### Electrophysiological recordings

Whole brains from 3–6 week old Sprague-Dawley (SD) male rats were placed in ice-cold external solution containing the following (in mM): 119 NaCl, 2.5 KCl, 2.5 CaCl_2_, 1.0 MgSO_4_, 1.25 NaH_2_PO_4_, 26.0 NaHCO_3_, 10 glucose, equilibrated with 95% O_2_ and 5% CO_2_ (pH 7.3–7.4) after capitation. Coronal slices (300 µm thick) containing the amygdala section cut with a DTK-1000 Microslicer (Dosaka, Kyoto, Japan) were continuously superfused in the external solution for at least an hour at room temperature (22-24°C) and used for experiments up to 6h after capitation. Slices were placed in the recoding chamber and perfused continuously with the external solution containing 100 µM picrotoxin to inhibit GABAergic transmission. Whole-cell recordings were made on the principal neurons in the lateral amygdala under infrared differential contrast visualization using Olympus BX50WI (Tokyo, Japan) and a charge-coupled device camera (Hamamatsu, Shizuoka, Japan) with an EPC-9 amplifier and Pulse v8.40 software (HEKA Elektronik, Germany). Patch electrodes (3–5 MΩ resistance) contained (in mM): 120 K-gluconate, 5 NaCl, 1 MgCl_2_, 0.2 EGTA, 10 HEPES, 2 MgATP, and 0.1 NaGTP (adjusted to pH 7.2 with KOH, 280-290 mOsm). Synaptic responses were filtered at 1 kHz and digitized at 5 kHz. If series resistance was changed by more than 20%, the experiments were discarded.

The two leads of the stimulus isolation unit (ISO-Flex, Jerusalem, Israel) were connected to the inside of the pipette and the external silver coat. Excitatory postsynaptic currents (EPSCs) or excitatory postsynaptic potentials (EPSPs) in the LA neuron were evoked at every 20 s by a bipolar stimulation of either the external capsule (cortical input) or the internal capsule (thalamic input) with the square current pulses (50-300 µA; 100 µs duration) using stimulator (NIHON KODEN) at room temperature unless indicated otherwise. In all LTP experiments, stimulus intensity was adjusted to produce synaptic responses with amplitude of 50-150 pA for voltage-clamp or 4-6 mV for current clamp mode, constituting 20-30% of maximum amplitude of EPSC or EPSP. Membrane potential was held constant at -70 mV throughout the experiments in voltage-clamp mode. After controlling the size of baseline EPSC or EPSP for 6 min, the several types of protocol for LTP induction were delivered. Summary LTP graphs were constructed by normalizing data in 60 s epochs to the mean value of baseline EPSC or EPSP (percentage baseline).

To examine the voltage dependence of EPSCs, 120 mM Cs-methane-sulfonate was used instead of K-gluconate. In experiments to block selectively postsynaptic NMDAR on principal neurons, cortical afferent fiber was stimulated repetitively while holding the postsynaptic cell at +30 mV after loading pyramidal neurons with NMDAR open-channel blocker, MK-801 (1 mM), via patch pipette under whole-cell configuration. In control, MK-801 was not included in the patch pipette. In estimating NMDAR-mediated currents, CNQX (20 µM) was applied to the external solution. In the experiment showing the effect of bath-applied chemicals on basal EPSC, the values at 15-20 min or 25-30 min (BAPTA-AM) after perfusion were compared to baseline.

BAPTA and MK-801 were dissolved directly into patch solution. Other chemicals were stored as frozen stock solutions in distilled water and diluted 1000-fold into bath solution immediately before use, with the exception of BAPTA-AM, SNAP, PTIO and ODQ, which were initially dissolved in DMSO and then diluted to bath solution.

### Behavioral procedures

4-6 weeks old male SD rats were maintained with free access to food and water under inverted 12/12 hours light/dark cycle. On the training day, rats were habituated in the conditioning chamber for a minimum of 15 min before onset of auditory fear conditioning. An initially neutral conditioned stimulus, a tone, lasting for 30 s (5 kHz, 80 dB), was terminated simultaneously with the unconditioned stimulus, foot-shock (0.5 mA, 0.5 s). The chamber was located in a sound-attenuating box. After two trials, rats were returned to their home cage. Rats were tested at 24-48h after conditioning. For testing, rats were placed in a novel environment where the same tone (60 s) was presented after a 10 min habituation period. Rats were considered to be freezing if no movement was detected for 3 s and the measure was expressed as a percentage of time spent freezing. Prior to behavioral training, rats were randomly assigned to two groups: conditioned (one paired with foot-shock) and control (tone alone). Immediately after test session, rats were used for electrophysiological recording.

### Data analysis

Data are presented as mean ± SEM. In assessing two different groups, two-tailed Student’s *t* test (paired or unpaired) was used for statistical analysis. Mann-Whitney U test was used to compare the amount of conditioned fear in the behavioral experiments. P<0.05 was considered statistically significant.

## Results

### Presynaptic NMDAR gates pre-LTP in cortical input

**Figure 1:**
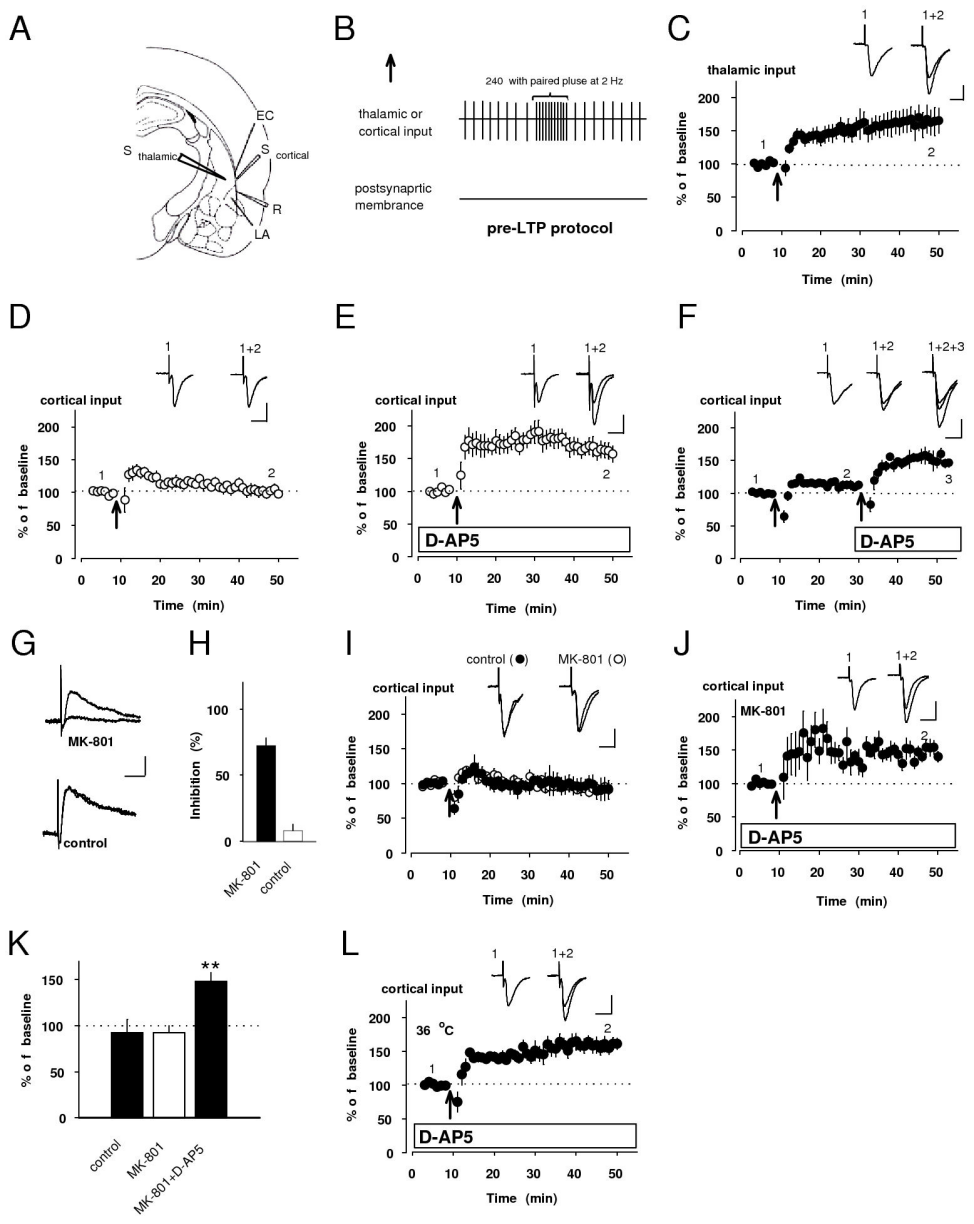
LTP in cortical input could be induced by presynaptic NMDAR blockage. **A**, Schematic representation of the slice preparation showing positions of stimulation (S_cortical_ and S_thalamic_) and recording electrode (R). S_cortical_ and S_thalamic_ were positioned to activate cortical and thalamic input, respectively. EC: external capsule. LA: the lateral nucleus of amygdala. **B**, A diagram illustrating the pre-LTP protocol: low-frequency stimulation of either thalamic or cortical input at holding potential of -70 mV. Detailed pattern, 240 pluses (2-Hz frequency) consisting of paired pulse stimulation (50 ms interval) is shown above the sign. **C**, EPSC in thalamic input was potentiated to 163 ± 20% of baseline by delivering pre-LTP protocol at the arrow (n=7, paired *t* test, p<0.05 versus baseline). **D**, The same protocol failed to produce LTP in cortical input (101 ± 6% of baseline, n=8, paired *t* test, p=0.86 versus baseline). **E**, However, it succeed to induce LTP with the addition of D-AP5 (50 µM) to the external solution (161 ± 12% of baseline, n=8, paired *t* test, p<0.01 versus baseline). Inserted traces (**C**-**E**) averages of 15 EPSCs recorded before (1) and 35-40 min after induction (2, arrow). **F**, Normalized LTP showing the effect of NMDAR on LTP induction at cortico-LA synapse. Insets: averaged EPSC recorded before (1) and after first (2, left arrow) and second inductions (3, right arrow). Scale bar (**C**-**F**) 50 pA, 20 ms. **G**, Superimposed first and last NMDAR-mediated EPSCs recorded at + 30 mV blocked by intracellular dialysis either with or without MK-801(control) in the presence of CNQX (20 µM). Scale bar: 50 pA, 50 ms. **H**, Summary showing the inhibition of NMDARs-mediated EPSCs with or without MK-801(control). **I**, Under control condition (without MK-801), EPSC in cortical input remained at 93 ± 8% of baseline (n=3, paired *t* test, p=0.99 versus baseline). Even after selective postsynaptic NMDAR blockade by MK-801, potentiation was still prevented (93 ± 8% of baseline, n=6, paired *t* test, p=0.37 versus baseline). Insets: superimposition of averaged EPSCs recorded before and after induction. **J**, Potentiation was readily induced under both the selective postsynaptic NMDAR blockade and bath-applied D-AP5. Insets: averaged EPSCs recorded before (1) and after (2) the induction. **K**, Summarized histogram showing quantification of results (**I**, **J**). **L**, EPSC in cortical input also potentiated to 158 ± 9% at physiological temperature, 35-36 °C (paired *t* test, n=5, p<0.01). Insets: average of 15 EPSCs recorded before (1) and after (2) induction (arrow). Scale bar (**I**, **J**, **L**) 50 pA, 20 ms. **p<0.01.

We classified principal neurons based on the pyramidal shape of their somata and their ability to show spike frequency adaptation upon depolarizing current injections in current-clamp mode after placing stimulating electrodes on either the internal or external capsular regions to activate thalamic or cortical inputs to the LA, respectively, in the acute slice preparation containing the LA [11,16-19] (Figure 1A). EPSCs were recorded under voltage-clamp mode at a holding potential of -70 mV in the presence of the GABA_A_ receptor antagonist, picrotoxin (100 µM), every 20 s (see *Electrophysiological recordings*). Both thalamic and cortical fibers, which converge on the same principal neurons of the LA, can be activated independently, as previously shown [16-19].

We confirmed that stimulation of thalamic input for 2 min with paired pulses (50 ms inter-pulse interval) at 2-Hz frequency without postsynaptic depolarization (pre-LTP protocol: Figure 1B) produced LTP (Figure 1C) in consistent with our previous study that LTP, observed at thalamo-LA synapse, depends on presynaptic KAR activation [16]. The above-described protocol is termed “pre-LTP protocol” (Figure 1B), because this protocol can lead to presynaptically induced LTP [16].

The same protocol delivered to cortical input, however, failed to induce LTP (Figure 1D). The observed differences was not due to differences in properties of synaptic transmission between cortical and thalamic inputs because cortico- and thalamo-LA synapses did not differ in either release probability or the size of postsynaptic responses to a single quantum of glutamate [18]. Surprisingly, EPSC in cortical input was significantly potentiated when pre-LTP protocol was delivered in the presence of the NMDAR antagonist, D-(-)-2-Amino-5-phosphonopentanoic acid (D-AP5, 50 µM) (Figure 1E).

To explore further the effect of NMDAR blockade on the mechanism for the induction of LTP in cortical input, we attempted to induce this type of potentiation in the same slice sequentially with or without D-AP5 (Figure 1F). We found that amplitude of the cortico-LA EPSC remained unchanged when pre-LTP protocol was delivered without D-AP5 (at first arrow), whereas it was significantly potentiated when the same protocol was delivered in the presence of D-AP5 (at second arrow, significant difference between EPSC magnitudes at 20 min after the induction at first and second arrows, n=5, paired *t* test, p<0.05). This indicates that the induction of LTP in cortical input may be suppressed through NMDAR-dependent mechanism. Postsynaptic NMDARs are not activated during the induction protocol because recorded LA neurons were voltage-clamped at -70 mV throughout the experiment, suggesting a possibility that NMDARs, suppressing LTP in cortical input, might be localized presynaptically. To address this possibility, we loaded recorded neurons with MK-801 (1 mM), an irreversible NMDAR open-channel blocker through the recording pipette, and stimulated repetitively cortical fibers at a potential of +30 mV to selectively block postsynaptic NMDAR [20,21] (see *Electrophysiological recordings*). The inhibition of NMDAR-mediated EPSC with MK-801 (n=5), 72 ± 6%, was significantly different from control (without MK-801, n=5), 8 ± 5% (unpaired *t* test, p<0.01, Figure 1G. H). As shown in Figure 1I, under conditions of the selective postsynaptic NMDAR blockade by MK-801, LTP in cortical input could not be observed (unpaired *t* test, p=0.99 versus control condition). However, it was rescued at a magnitude of 148 ± 9% of baseline by additional D-AP5 to the external solution (n=3, paired *t* test, p<0.05 versus baseline; Figure 1J), which was also significantly different from its pre-D-AP5 value (unpaired *t* test p<0.01; Figure 1K), suggesting the possibility that presynaptic NMDAR activation might suppress the induction of LTP at cortico-LA synapse. This potentiation was also induced at physiological temperature, 35-36 °C (Figure 1L), indicating that this type of synaptic plasticity in cortical input might have the physiological significance.

**Figure 2:**
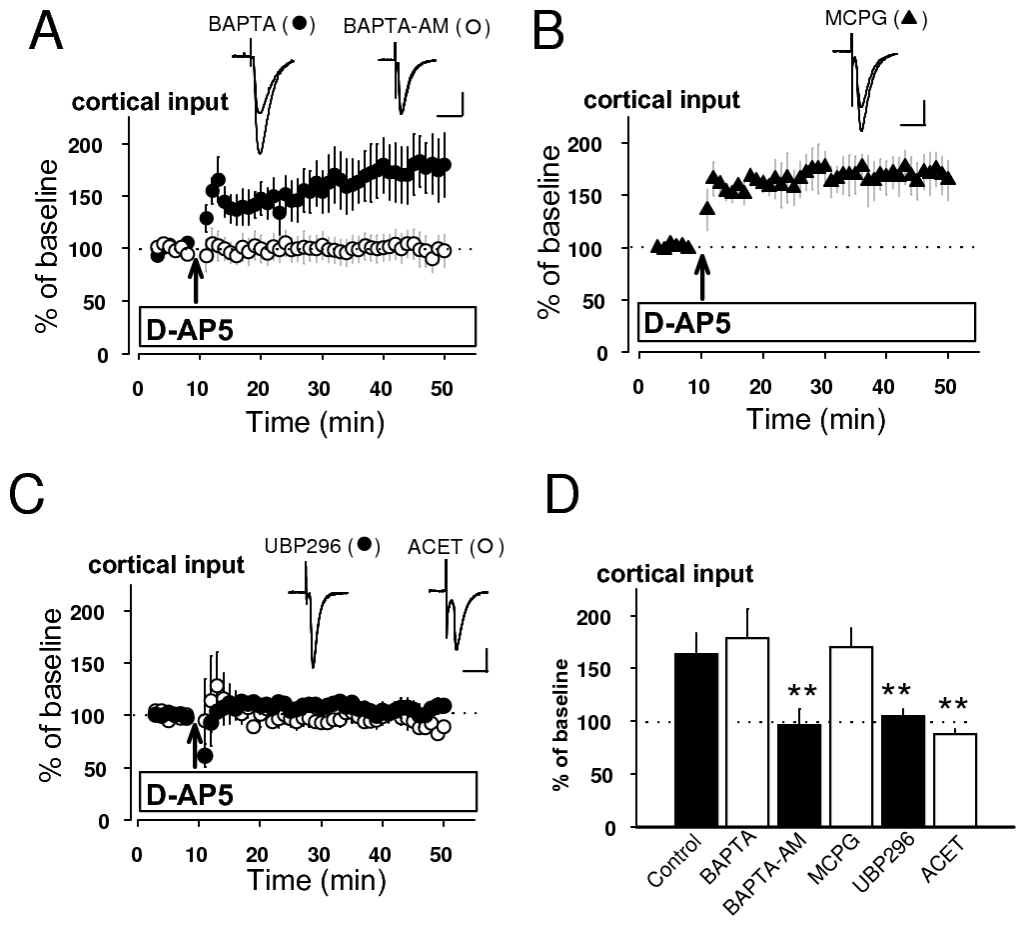
LTP in cortical input also depends on presynaptic KAR. **A**-**C**, Graphs summarizing LTP experiments showing the effect of Ca^2+^ chelators (**A**: BAPTA, BAPTA-AM), metabotropic glutamate receptor antagonist (**B**: MCPG) and selective KAR antagonists (**C**: UBP296, ACET) on the induction of LTP in cortical input. The respective insets show superimposition of averaged EPSCs recorded before and after the induction in the presence of each drug. Scale bars (**A**-**C**) 50 pA, 20 ms. **D**, Quantification of pharmacological analysis (**A**-**C**). Control was obtained from the result in Figure 1E. LTP in cortical input was blocked by BAPTA-AM (97 ± 15% of baseline, n=8, unpaired *t* test, p<0.01 versus control), but not by BAPTA (179 ± 27% of baseline, n=6, unpaired *t* test, p=0.53 versus control). This potetiation was resistant to MCPG (170 ± 18% of baseline, n=6, unpaired *t* test, p=0.68 versus control), but it was prevented by either UBP296 (105 ± 7% of baseline, n=5, unpaired *t* test, p<0.01 versus control) or ACET (88 ± 5% of baseline, n=5, unpaired *t* test, p<0.01 versus control). **p<0.01.

We then characterized the requirements for this type of LTP in cortical input. Unlike conventional pairing-induced LTP [11,18,19], this potentiation was not blocked by the inclusion of a high concentration (20 mM) of the fast Ca^2+^ chelator 1,2-bis (o-aminophenoxy) ethane-N,N,N',N'-tetraacetic acid (BAPTA) in the recording pipette solution (Figure 2A, D), suggesting that its induction does not require postsynaptic Ca^2+^ influx. However, pretreatment of slices with the external solution containing a cell-permeable Ca^2+^ chelator, BAPTA-AM (50 µM), blocked this type of LTP (Figure 2A, D), indicating that presynaptic Ca^2+^ influx might be implicated in its induction process. In this experiment, DMSO and probenecid were used to dissolve and prevent extrusion of BAPTA-AM, respectively. Under the presence of both DMSO (0.1% of the external solution) and probenecid (1 mM), EPSCs at cortico-LA synapse were also substantially potentiated to 150 ± 7% of baseline by delivering pre-LTP protocol (n=3, unpaired *t* test, p=0.57 versus control in Figure 1E: data not shown), indicating that neither DMSO nor probenecid affects LTP induction. EPSC amplitude insignificantly decreased by 15 ± 6% (n=8: data not shown) at 30 min after switching to bath solution containing BAPTA-AM (50 µM), suggesting that BAPTA-AM does not affect basal synaptic transmission. Before LTP experiments acute slices were treated with BAPTA-AM for at least 30 min for stabilization.

We next examined possible contributions of non-NMDA glutamate receptors with known ability to mediate increases in intracellular Ca^2+^ concentration to the induction process. LTP in cortical input, observed by D-AP5, was insensitive to a non-selective mGluR antagonist, α-Methyl-4-carboxyphenylglycine (MCPG) (500 µM) (Figure 2B, D). GluR5, one of the subunits of KAR, highly expressed in the amygdala [22], has been shown to mediate certain forms of synaptic plasticity [16,17,22]. In agreement with our previous finding that pre-LTP in thalamic input, induced by the same low-frequency stimulation without D-AP5, depends on presynaptic KAR activation [16], LTP in cortical input, observed by application of D-AP5, was completely blocked by a selective antagonist of the GluR5 subunit-containing KAR, UBP296 (1 µM) or (S)-1-(2-Amino-2-carboxyethyl)-3-(2-carboxy-5-phenylthiophene-3-yl-methyl)-5-methylpyrimidine-2, 4-dione, ACET (0.5 µM) (Figure 2C, D). These findings suggest that cortico-LA synapses can undergo LTP mediated by activation of presynaptic KAR and subsequent presynaptic Ca^2+^ influx. Hence, both thalamo- and cortico-LA synpases have the same ability to produce pre-LTP by activation of presynaptic KAR.

### Presynaptic NMDAR exerts suppression of pre-LTP by NO-cGMP signaling

**Figure 3:**
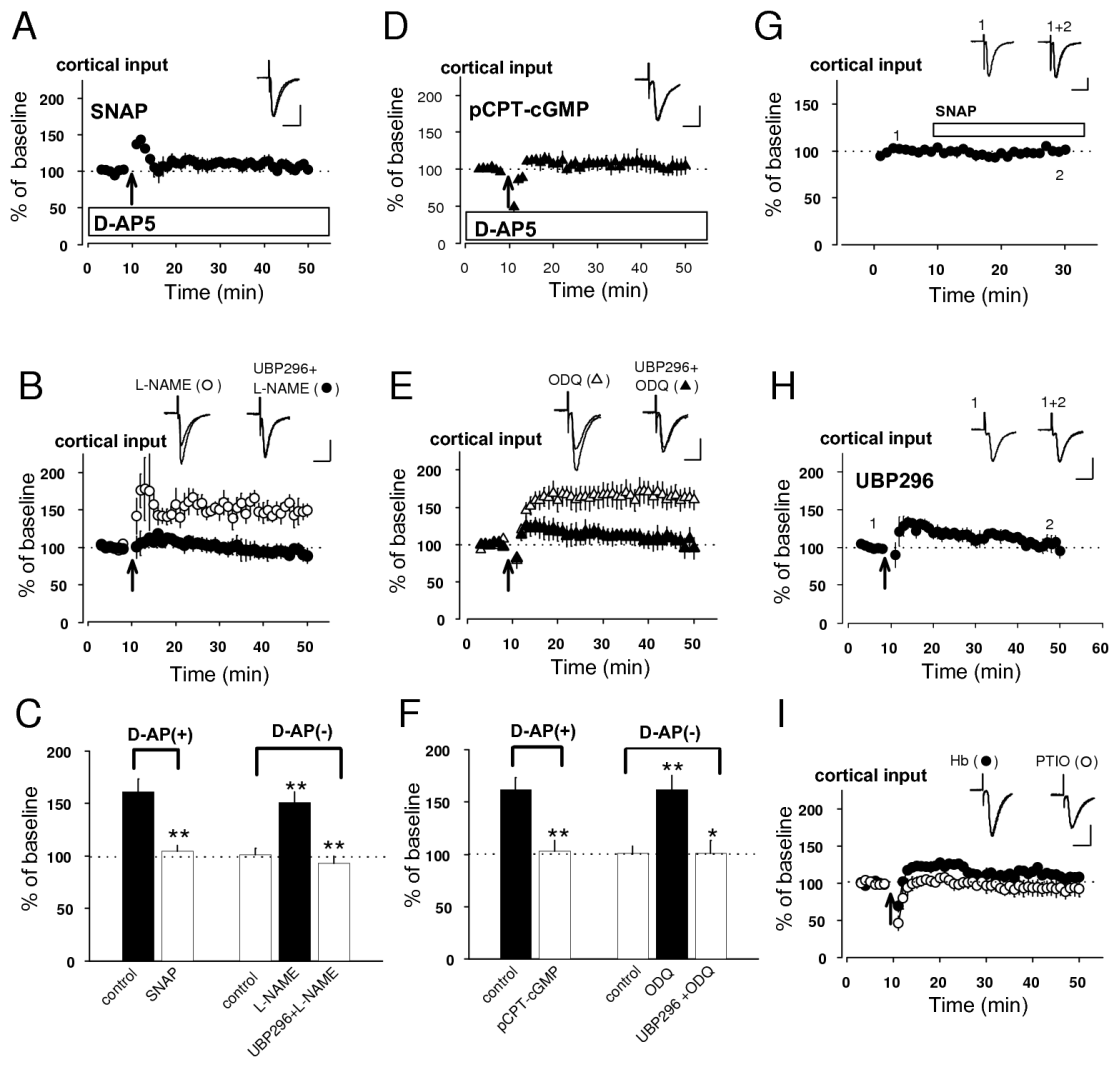
NO-cGMP signaling suppresses pre-LTP. **A**, **B**, Effect of SNAP (**A**), L-NAME alone and L-NAME + UBP296 (**A**, **B**) on the pre-LTP induction. Insets: superimposition of averaged EPSCs before and after the pre-LTP induction. **C**, Histogram showing quantification of the experimental results (**A**, **B**). Control, *left* and *right*, was obtained from the result in Figure 1E and 1D, respectively. Pre-LTP was prevented by bath applied SNAP. L-NAME produced synaptic potentiation despite the absence of D-AP5 and this was blocked by addition of UBP296. **D**, **E**, Effect of pCPT-cGMP (D), ODQ alone and ODQ + UBP296 (**E**) on the pre-LTP induction. Insets: superimposition of averaged EPSCs before and after the induction. *F*, Histogram showing the results shown in **D**, **E**. Control, *left* and *right*, was obtained from the result in Figure 1E and 1D, respectively. EPSCs were remained by the addition of pCPT-cGMP to bath. ODQ resulted in substantial potentiation despite the absence of D-AP5 (162 ± 13% of baseline, n=7, unpaired *t* test, p<0.01 versus control in Figure **1D**) and its potentiation was also blocked by UBP296 (100 ± 0% of baseline, n=5, unpaired *t* test, p<0.01 versus its pre-UBP296 value). **G**, **H**, Effect of exogenous (**G**) and endogenous (**H**) NO on basal transmission. **G**, Bath application of SNAP did not have any significant effect on basal transmission. Insets: averaged EPSCs recorded before (1) and 25-30 min after (2) application of SNAP. **H**, EPSCs were not affected by pre-LTP protocol in the presence of UBP296. Insets: averages of EPSCs before (1) and after (2) induction. **I**, EPSCs were not potentiated in the presence of NO scavenger, either Hb or PTIO. *p<0.05 **p<0.01 Scale bar: 50 pA, 20 ms.

It has been previously reported that NMDAR activation may suppress the induction of hippocampal LTP through NO-dependent mechanism [23]. NO production has widely been known to be dependent on postsynaptic NMDARs activation and subsequent activation of NOS, but a recent study using electron microscopy and immunochemistry has reported that NOS is expressed in axon terminals forming symmetric and asymmetric synapses onto spines of cells in the amygdala, suggesting that both excitatory and inhibitory terminals might contain NOS [13]. In this scenario, we hypothesized that NO, produced as a result of presynaptic NMDAR activation, may suppress the induction of pre-LTP. Consistent with this notion, pre-LTP in cortical input, observed under conditions of NMDAR blockade (Figure 1E), was prevented by a NO donor, *S*-nitroso-*N*-acetylpenicillamine (SNAP, 200 µM) in the external solution (105 ± 5% of baseline, n=5, unpaired *t* test, p<0.01 versus control in Figure 1E: Figure 3A, C). Moreover, robust LTP could be induced in the presence of a NOS inhibitor, Nω-nitro-L-arginine (L-NAME, 200 µM) even when the external solution did not contain D-AP5 (151 ± 10% of baseline, n=5, unpaird *t* test, p<0.01 versus control in Figure 1D: Figure 3B, C). This potentiation, observed by L-NAME, was blocked by UBP296 (93 ± 7% of baseline, n=3, unpaired *t* test, p<0.01 versus its pre-UBP296 value), proving that it was KAR-dependent, putative pre-LTP.

We also tested whether exogenous and endogenous NO could affect basal synaptic transmission. The EPSC amplitude remained unchanged in the course of bath application of SNAP in the same concentration used in the above-mentioned LTP experiments (100 ± 3% of baseline at 25-20 min after bath-applied SNAP, n=5, paired *t* test, p=0.94 versus baseline: Figure 3G), indicating the lack of direct effects of exogenous NO on basal synaptic transmission. We then explored the effects of endogenous NO on basal EPSCs by monitoring EPSCs after application of UBP296 blocking pre-LTP induction. Under these conditions, pre-LTP protocol would trigger endogenous release of NO, but not synaptic plasticity. EPSCs remained unchanged by pre-LTP protocol in the presence of UBP296 (103 ± 19% of baseline, n=5, paired *t* test, p=0.81 versus baseline; Figure 3H). Taken together, these findings demonstrate that NO may influence the induction process without affecting basal synaptic transmission.

NO has been shown earlier to modulate synaptic efficacy through a cGMP-dependent mechanism [24]. In our experiments, an analogue of cGMP, pCPT-cGMP (100 µM), suppressed pre-LTP when added to the external solution (103 ± 10% of baseline, n=4, unpaired *t* test, p<0.05 versus control in Figure 1E: Figure 3D, F). Conversely, pretreatment of slices with an inhibitor of NO-sensitive soluble guanylyl cyclase, 1*H*- [1,2,4] oxadiazolo[4,3-a] quinoxalin-1-one (ODQ, 10 µM), resulted in KAR-dependent potentiation despite the absence of D-AP5 in external medium (Figure 3E, F). These results indicate that cGMP signaling may contribute to the suppression of pre-LTP.

Bath application of a membrane-impermeable scavenger of NO, either hemoglobin (Hb, 100 µM) or 2-phenyl-4,4,5,5-tetramethylimidazoline-1-oxyl-3-oxide (PTIO, 300 µM) failed to block the NMDAR-induced prevention of pre-LTP in cortical input (Hb, 106 ± 5% of baseline, n=6, unpaired *t* test, p=0.59 versus control in Figure 1E, PTIO, 92 ± 10% of baseline, n=6, unpaired *t* test, p=0.35 versus control in Figure 1E: Figure 3I), indicating that the suppression of pre-LTP in cortical input did not require the role of NO in extracellular space.

### Presynaptic NMDAR in cortical input heterosynaptically suppressed pre-LTP in thalamic input by NO diffusion

A recent imaging study provided evidence that cortical and thalamic afferents could converge on the same dendrite branch of the LA projection neuron, forming active synapses on spines, which could be as close as less than 5 µm [25]. In the cerebellar cortex, NO was shown to spread over one hundred micrometers and trigger synaptic plasticity at non-activated synapses on neighboring neurons [2]. These reports led us to the assumption that NO, released from the cortico-LA synapses, could suppress pre-LTP in thalamic input in a heterosynaptic manner.

**Figure 4:**
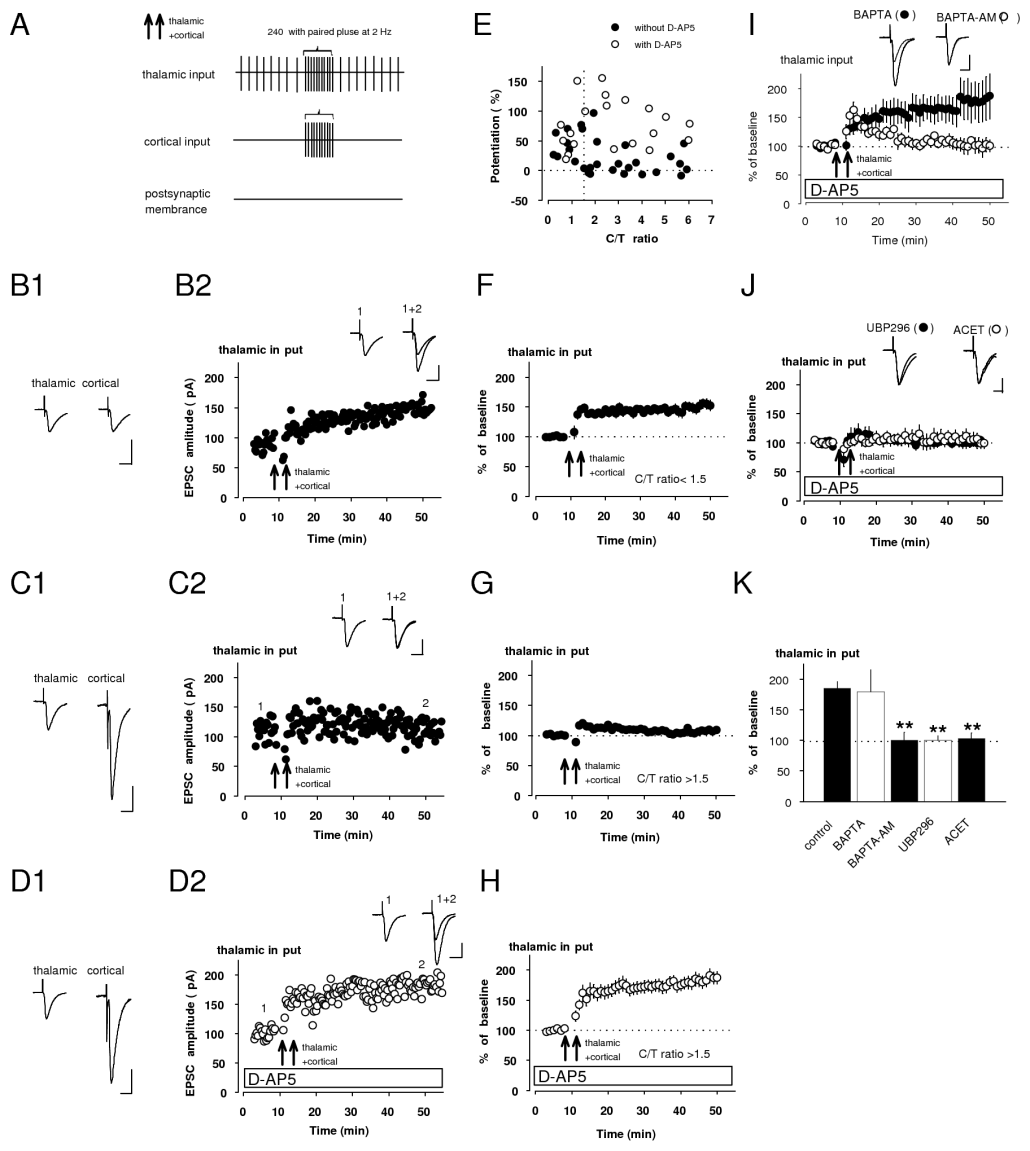
Pre-LTP in thalamic input depends on synaptic activity at cortico-LA synapses. **A**, Schematic representation of the induction protocol used in this experiment. Double arrows indicate the induction protocols consisting of paired stimulation of both thalamic and cortical inputs without postsynaptic depolarization. **B**, **C**, An individual experiment (**B2, C2**) investigating whether LTP could be induced or not by the induction when the amplitude of cortical EPSC is smaller (**B1**) or larger (**C1**) than that of thalamic. **D**, An individual LTP experiment (**D2**) showing effect of D-AP5 on the non-potentiated EPSC (**C2**). Insets (**B1-D1**): EPSCs in both thalamic and cortical inputs before the induction. Insets (**B2-D2**): averaged EPSCs before (1) and after (2) delivery of the induction (double arrows). Scale bar (**B**-**D**) 50 pA, 20 ms. **E**, Relationship between C/T ratio (before the induction) and the magnitude of LTP in thalamic input induced the induction with (open circles) or without D-AP5 (closed circles) in the bath solution. Each symbol represents a separate experiment. **F**-**H**, Summary graphs of LTP experiments (**B**-**D**) EPSCs observed with C/T in a range below 1.5 resulted in robust potentiation (**F**: 152 ± 8% of baseline, n=10, paired *t* test, p<0.01 versus baseline). When the data were collected in a range of C/T > 1.5, lesser LTP was observed (**G**, 110 ± 4% of baseline, n=19, paired t test, p=0.045 versus baseline). In the same range of C/T, the EPSCs were significantly potentiated by addition of D-AP5 (**H**, 185 ± 11% of baseline, n=13, paired *t* test, p<0.01 versus baseline). **I**, This observed potentiation was resistant to BAPTA (20 mM) (180 ± 36% of baseline, n=6, p=0.85 versus control by unpaired *t* test), but was blocked by BAPTA-AM (50 µM) (100 ± 13% of baseline, n=5, p<0.01 versus control by unpaired *t* test). **J**, LTP_ct_ was completely blocked by either UBP296 (1 µM) (100 ± 7% of baseline, n=4, p<0.01 versus control by unpaired *t* test) or ACET (0.5 µM) (103 ± 10% of baseline, n=5, p<0.01 versus control by unpaired *t* test). Insets: superimposed averaged EPSCs recorded before and after the induction with application of each drug. Scale bars: 50 pA, 20 ms. **K**, Quantification of pharmacological experiment results (**H**, **I**, **J**). Control was obtained from the result in Figure **4H**. **p<0.01; unpaired *t* test.

To test this notion, we investigated how EPSC in thalamic input was potentiated by delivering protocols for simultaneous inductions at both thalamic and cortical pathways (Figure 4A). This protocol resulted in substantial potentiation of the EPSC in thalamic input (Figure 4B2) when the EPSC amplitude in cortical input was lesser than that in thalamic input (Figure 4B1). When the amplitude of cortico-LA EPSC exceeded the amplitude of thalamo-LA EPSC (Figure 4C1), no LTP in thalamic input was observed (Figure 4C2). LTP could be observed, however, under the same condition (Figure 4D1) after an addition of D-AP5 to the external solution (Figure 4D2). Then we plotted the magnitude of LTP in thalami input, measured in many individual experiments, as a function of the cortical/thalamic EPSC amplitude ratios (cortical EPSC amplitude was divided by thalamic EPSC amplitude: C/T ratio) either in the absence or presence of D-AP5 (Figure 4E). For C/T < 1.5, all EPSCs were potentiated and this observation was unchanged by an addition of D-AP5 to the bath solution. When C/T ratio was greater than 1.5 (C/T > 1.5), in most experiments, most EPSCs largely remained unchanged following the induction, but significant LTPs were observed in the presence of D-AP5. In the experiments with C/T < 1.5 (Figure 4F), the magnitude of LTP was significantly larger compared to those with C/T > 1.5 (unpaired *t* test, p<0.01) (Figure 4G), leading to the notion that observed potentiation in thalamic input depends on the size of synaptic responses at cortico-LA synapses. In the experiments with C/T > 1.5, LTP was rescued by bath-applied D-AP5 (LTP_ct_; Figure 4H) (unpaired *t* test, p<0.01 versus experiments without D-AP5). This suggests a possibility that the induction of LTP at thalamo-LA synapses, may depend on synaptic activity at cortico-LA synapse via NMDAR-mediated mechanisms.

Because LTP_ct_, resulting from co-activation of cortical and thalamic afferents, was induced by glutamate both released from thalamic fibers and diffused from cortical terminals during co-stimulation of the convergent pathways under NMDAR blockade, we compared the mechanism for LTP_ct_ induction to that for pre-LTP induced by stimulation of thalamic input alone. Similar to pre-LTP in thalamic input [16], LTP_ct_ (control) was blocked by bath-applied BAPTA-AM, but not by postsynaptic BAPTA loading (Figure 4I, K). Either UBP296 or ACET prevented LTP_ct_ (Figure 4J, K). Thus, LTP_ct_ (control) was mechanistically similar to pre-LTP in thalamic input reported in our previous work [16]. In other words, presynaptic NMDAR activation in cortical input might suppress the induction of pre-LTP in thalamic input in heterosynaptic manner.

**Figure 5:**
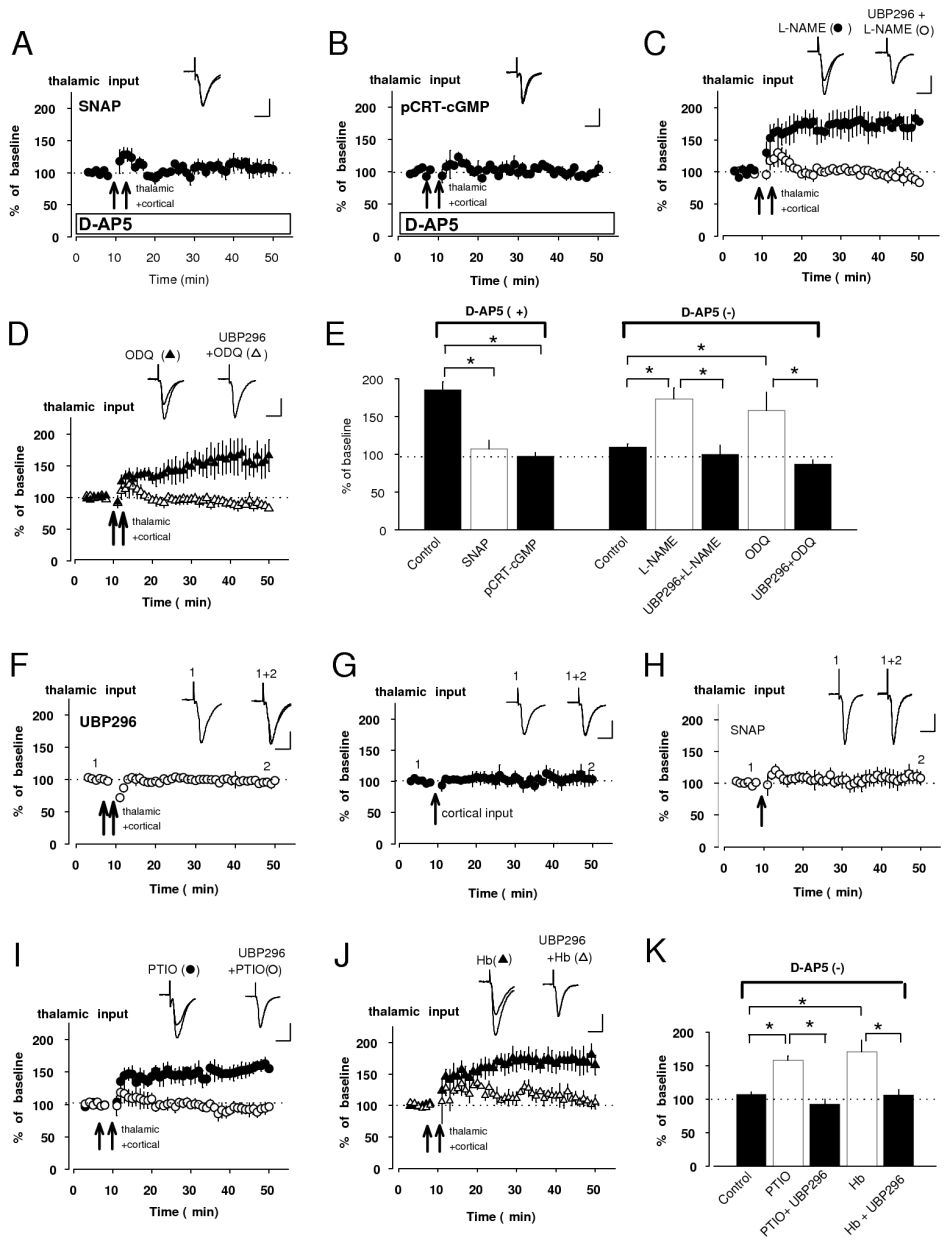
Pre-LTP at thalamic input can be also suppressed by NO diffusion. **A**, **B**, LTP_ct_ was prevented by either SNAP (**A**, 107 ± 11% of baseline, n=4, unpaired *t* test, p<0.01 versus control) or pCPT-cGMP (**B**, 97 ± 6% of baseline, n=3, unpaired *t* test, p<0.01 versus control). **C**, **D**, Either L-NAME (**C**) or ODQ (**D**) produced robust potentiation (L-NAME: 173 ± 15% of baseline, n=3, unpaired *t* test, p<0.01 versus control; ODQ: 158 ± 23% of baseline, n=6, unpaired *t* test, p<0.01 versus control) in the absence of D-AP5. These potentiations were blocked by UBP296 (L-NAME+UBP296: 100 ± 12% of baseline, n=4, unpaired *t* test, p<0.05 versus its pre-UBP296 value; ODQ+UBP296: 87 ± 5% of baseline, n=4, unpaired *t* test, p<0.05 versus its pre-UBP296 value). Insets (**A**-**D**); superimposition of averaged EPSCs recorded before and after co-stimulation induction (double arrow). Scale bar: 50 pA, 20 ms. **E**, Histogram showing quantifications of results (**A**-**D**). Control, *left* and *right*, was obtained from the result in Figure **4H, G**, respectively. **F**, EPSC was unchanged with the induction consisting of paired thalamic with cortical inputs in the presence of UBP296 (95 ± 3% of baseline, n=3, paired *t* test, p=0.21 versus baseline). **G**, EPSC in thalamic input was not affected by pre-LTP protocol delivered at cortical input alone (105 ± 11% of baseline, n=4, paired *t* test, p=0.66 versus baseline). **H**, Exogenous SNAP completely blocked pre-LTP in thalamic input (110 ± 11% of baseline, n=5, paired *t* test, p=0.43 versus baseline). Traces (**F**-**H**) averages of EPSCs recorded before (1) and after (2) each induction. **I**, **J**, Robust synaptic potentiation was induced by either PTIO (**I**, 158 ± 6% of baseline, n=4, unpaired *t* test, p<0.01 versus control) or Hb (**J**, 170 ± 18% of baseline, n=4, unpaired *t* test, p<0.01 versus control) despite the absence of D-AP5. Potentiations observed by these scavengers were completely blocked by UBP296 (**I**, PTIO+UBP296: 93 ± 8% of baseline, n=5, unpaired *t* test, p<0.01 versus its pre-UBP296 value. **J**, Hb+UBP296:106 ± 9% of baseline, n=3, unpaired *t* test, p<0.05 versus its pre-UBP296 value). **K**, Quantification of experimental results (**F**, **G**). Control was obtained from the result in Figure **4G**. Scale bar: 50 pA, 20 ms. **p<0.01.

We also could detect NMDAR-mediated suppression of pre-LTP when C/T ratio was greater than 1.5. Next we investigated the mechanism of this suppression. Either SNAP or pCPT-cGMP completely blocked LTP_ct_, which was induced in the range of C/T ration more than 1.5 (C/T > 1.5) (Figure 5A, B). SNAP in the same concentration had practically no effect on basal synaptic transmission in thalamic input (100 ± 10% of baseline, n=9, paired *t* test, p=0.98 versus baseline: data not shown). Robust LTP_ct_ was observed by either L-NAME or ODQ instead of D-AP5 in the range of more than 1.5 (C/T > 1.5), and it was blocked by additional UBP296, indicating that this potentiation was also KAR-dependent (pre-LTP; Figure 5C, D, E).

We checked the effect of endogenous NO, produced by activation of presynaptic NMDAR localized in cortical terminals, on basal transmission in thalamic input. The amplitude of basal EPSCs were not affected by endogenous NO, triggered by delivering LTP induction protocol at both thalamic and cortical inputs simultaneously in the presence of UBP296 to block the induction of LTP_ct_ (Figure 5F). We monitored EPSCs at thalamic input in a condition where cortical input was largerer than thalamic amplitude to explore the heterosynaptic effect of endogenous NO on basal synaptic transmission. The EPSC in thalamic input also was not influenced by endogenous NO production via delivering pre-LTP protocol at cortical input alone (Figure 5G). These results suggest that endogenous NO did not affect basal synaptic transmission at thalamo-LA synapse.

We explored the direct effect of exogenous NO on the induction of pre-LTP in thalamic input. This potentiation was blocked by exogenously applied SNAP (Figure 5H) (unpaired *t* test, p<0.05 versus control LTP without SNAP, see Figure 1C). Either PTIO (300 µM) or Hb (100 µM), promoted this type of potentiation despite unblocked NMDARs, and these effects were completely reversed by the addition of UBP296 (Figure 5I, J, K). These findings indicate that blockade of pre-LTP by NO rereleased from cortico-LA synapse needs the diffusion of NO in the extracellular space, providing support to the idea that there may be the interaction between presyaptic sites of cortical and thalamic pathways via heterosynaptic transmission of NO.

### Different contributions of pre- and post-LTPs in encoding fear memory

**Figure 6:**
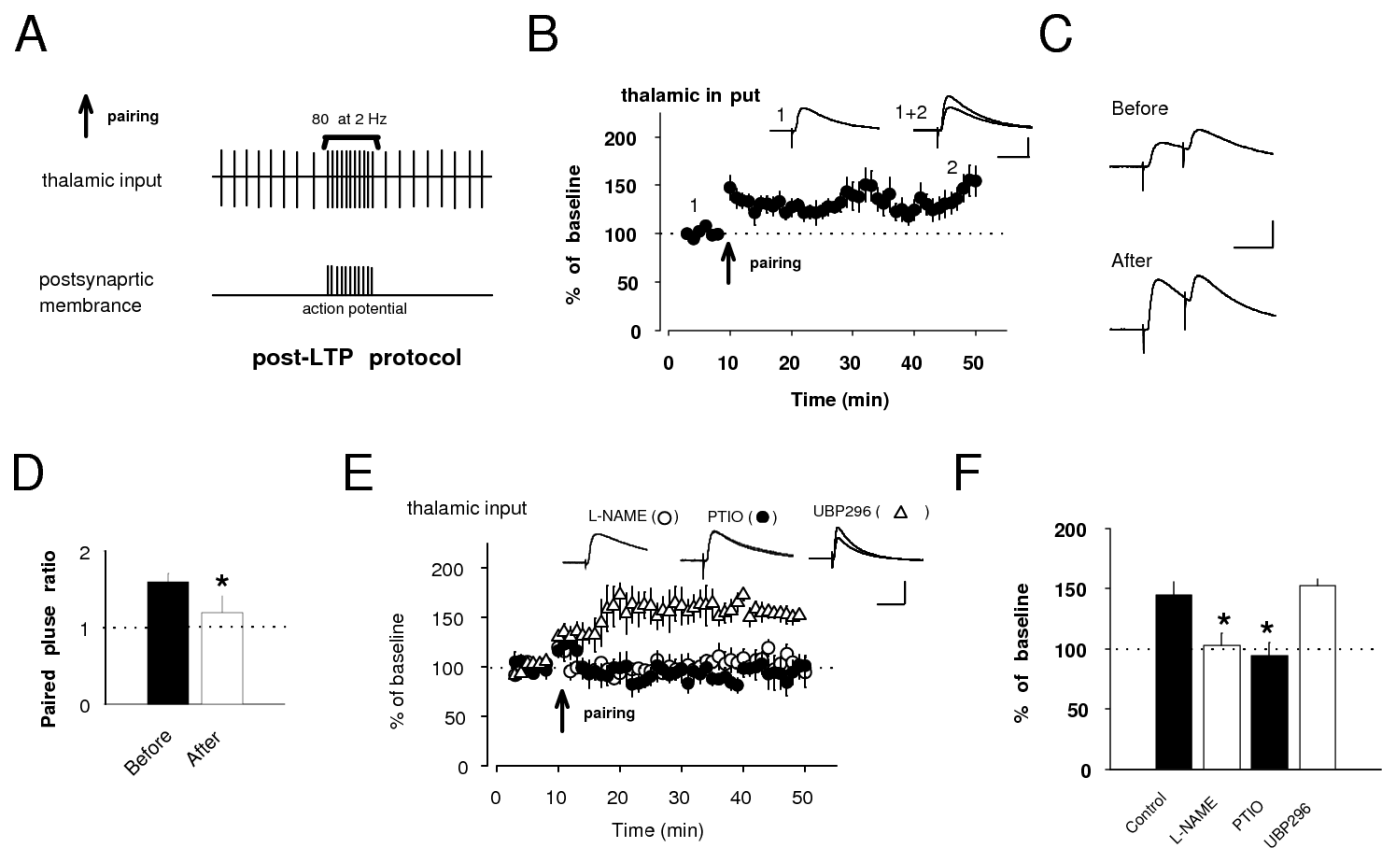
Post-LTP in thalamic input requires NO signaling. **A**, A diagram illustrating the post-LTP protocol consisting of 80 pairing stimulation at thalamic input with action potential with the delay of 4-8 ms at 2 Hz. Detailed pattern is described above the sign. **B**, EPSC in thalamic input was potentiated to 145 ± 12% of baseline by delivering the post-LTP protocol (n=4, paired *t* test, p<0.05 versus baseline). **C**, **D**, Examples of the EPSC (**C**) and summary of paired-pulse ratio (**D**) evoked by paired-pulse stimulation with 50 ms interval at thalamo-LA synapse before and after post-LTP protocol. **E**, The induction of post-LTP was not prevented by bath application of UBP296 (152 ± 6% of baseline, n=4, paired *t* test, p<0.01). Bath application of L-NAME and PTIO completely blocked post-LTP (L-NAME: 103 ± 10% of baseline, n=4, unpaired *t* test, p<0.05 versus control. PTIO: 94 ± 11% of baseline, n=4, unpaired *t* test, p<0.05 versus control). **F**, Quantification of experimental results in **E**. Control was obtained from the result in B. Scale bar: 5 mV, 50 ms. *p<0.05.

A previous study has shown that NO signaling has a vital role in the induction of conventional LTP in thalamic input to the LA and the acquisition of conditioned fear [13]. Before exploring the role of NO in the conventional (pairing protocol-induced) LTP in thalamic input, we confirmed that pairing of 80 presynaptic pulses, delivered to thalamic pathway at 2 Hz with action potentials evoked in the postsynaptic cell with a 4-8 ms delay from the onset EPSP by short depolarizing current injections through the recording electrodes (post-LTP protocol: Figure 6A) in current-clamp mode could lead to LTP (Figure 6B) in consistent with previous study [18]. This form of LTP, previously known to depend on the influx of Ca^2+^ via postsynaptic NMDAR activation (post-LTP) [18], was insensitive to KAR antagonist, UBP296 (Figure 6E, F), indicating that its form of LTP might be dependent on NMDAR activity. It was prevented by bath application of either L-NAME or PTIO (Figure 6E), confirming that post-LTP might be mediated postsynaptic NMDAR and consequent NO production. Post-LTP induction also led to a substantial depression of paired pulse ratio (PPR) of EPSPs in thalamic input. PPR, 1.20 ± 0.21, after the induction, was significantly different from PPR, 1.60 ± 0.11, before the induction (n=4, paired *t* test, p<0.05: Figure 6C, D), implying presynaptic involvement in post-LTP at terminal of thalamic input [26]. These results indicate that NO, produced in postsynaptic cell by NMDAR activation, might diffuse as a retrograde factor to the presynaptic terminal, leading to enhanced transmitter release.

Direct infusion of either NMDAR antagonist [9] or NOS inhibitor [13] into rat amygdala bilaterally impaired fear conditioning, suggesting that NMDAR-induced NO signaling might contribute to encoding of fear memory. A recent study has also demonstrated that pharmacological pre-training KAR blockade in the LA suppresses fear conditioning [17], suggesting that KAR-dependent processes might contribute to encoding fear memory. Moreover, it has previously been demonstrated that fear conditioning induced LTP-like enhancements of synaptic strength in conditioned stimulus pathways, which occluded LTP induced with electrical stimulation in slices containing the LA [11,17].

**Figure 7:**
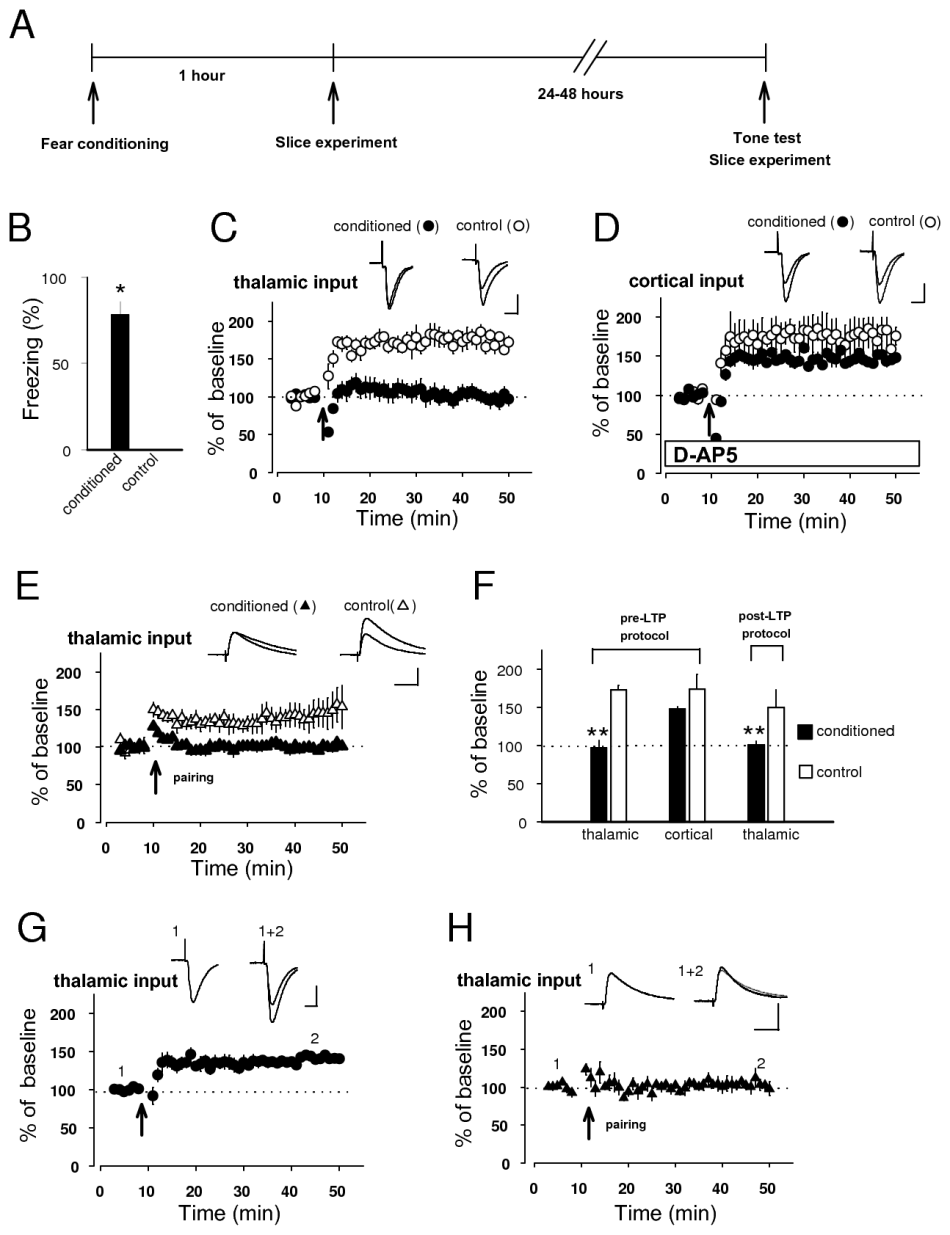
Fear conditioning occludes synaptic plasticity at thalamic, but not cortical input. **A**, Illustration depicting the experimental design. **B**, There was a significant difference in freezing in response to the conditioned tone between conditioned (78 ± 8%, n=16) and control rats (0 ± 0%, n=8: Mann-Whitney *U* test, p<0.01). **C**, **D**, Graph summarizing pre-LTP in thalamic (**C**) and cortical (**D**) input in conditioned and control rats at 24-48 h after fear conditioning. Pre-LTP in thalamic input was not induced in conditioned rats, whereas it was fully induced in control rats. The same type of synaptic plasticity in cortical input were readily induced in both conditioned and control rats (conditioned: 148 ± 2% of baseline, n=6, control: 173 ± 10% of baseline, unpaired *t* test, p=0.25). **E**, Summary of post-LTP experiment in conditioned and control rats at 24-48 h after fear conditioning. Post-LTP in thalamic input was occluded in the slice of conditioned rats, whereas it was significantly induced in the slice of control rats. **F**, Histogram showing quantification of results (**C**-**E**). ** p<0.01. **G**, **H**, Normalizing LTP experiment showing pre-LTP (**G**) and post-LTP (**H**) at thalamo-LA synapse in conditioned rats at 1h after fear conditioning. Pre-LTP was significantly induced, whereas post-LTP was fully occluded. Insets: superimposition of averaged EPSCs or EPSPs recorded before and after induction in these groups. Scale bar: 50 pA, 20 ms for voltage-clamp mode and 5 mV, 20 ms for current-clamp mode.

To correlate memory of conditioned fear with the induction of pre-LTP in both thalamic and cortical inputs or post-LTP in thalamic input, we tested these forms of synaptic plasticity in acute brain slices prepared from fear-conditioned or control (tone alone) rats (see *Behavioral Procedures*). Fear-conditioned rats froze significantly more in response to the conditioned tone at 24-48 hours (h) post-conditioning compared to control rats. Shortly after the fear memory test, we performed whole-cell recording from pyramidal neurons of the acute slice from both rats in *ex vivo* experiments. We found that pre-LTP in thalamic input was prevented in slices from fear-conditioned rats, whereas it was readily induced in slices from control (conditioned: 97 ± 9% of baseline, n=5, control: 172 ± 6% of baseline, n=4, unpaired *t* test, p<0.05; Figure 7C). This finding indicates that pre-LTP in thalamic input was occluded following the acquisition of fear memory, suggesting that pre-LTP-like mechanisms may contribute to encoding the fear memory trace in consistent with the notion that KAR-dependent plasticity may be linked to fear conditioning [17]. However, pre-LTP in cortical input was not occluded in the slice from either conditioned or control rats (Figure 7D). Regarding post-LTP in thalamic input, we did detect significant difference in their magnitude between conditioned and control rats (conditioned: 100 ± 5% of baseline, n=7, control: 150 ± 22% of baseline, n=4, unpaired *t* test, p<0.05; Figure 7E, F), suggesting that this form of synaptic plasticity in thalamic input also might be an essential cellular mechanism contributing to encoding of conditioned fear.

Short-term memory (STM) and long-term memory (LTM) of learned fear, measured at 1h and 24 h, respectively, after fear conditioning training were previously estimated to explore the mechanism underlying fear conditioning [13], but, the link between STM and synaptic plasticity has not been explored. Consolidation of fear, which is stabilized over several hours after fear conditioning, may critically depends on synaptic enhancement in the LA. However, consolidation may become labile temporarily after tone test reactivating fear memory and can be stabilized again (reconsolidation). Because we explored a close link between consolidation process of fear memory and synaptic plasticity, we investigated synaptic plasticity of conditioned animals without tone test at 1 hour after fear conditioning.

We investigated whether and how pre- and post-LTPs in thalamic input are induced or occluded in the slice prepared from conditioned rats immediately (1 h) after fear conditioning. EPSCs were significantly potentiated to the magnitude of 141 ± 2% of baseline by delivering the pre-LTP protocol (n=5, paired *t* test, p<0.05 versus baseline; Figure 7G), whereas EPSPs were remained to be 100 ± 5% of baseline by the post-LTP protocol (n=7, paired *t* test, p=0.9 versus baseline; Figure 7H), indicating that post-LTP could be formed more rapidly than pre-LTP is after or during fear conditioning.

**Figure 8:**
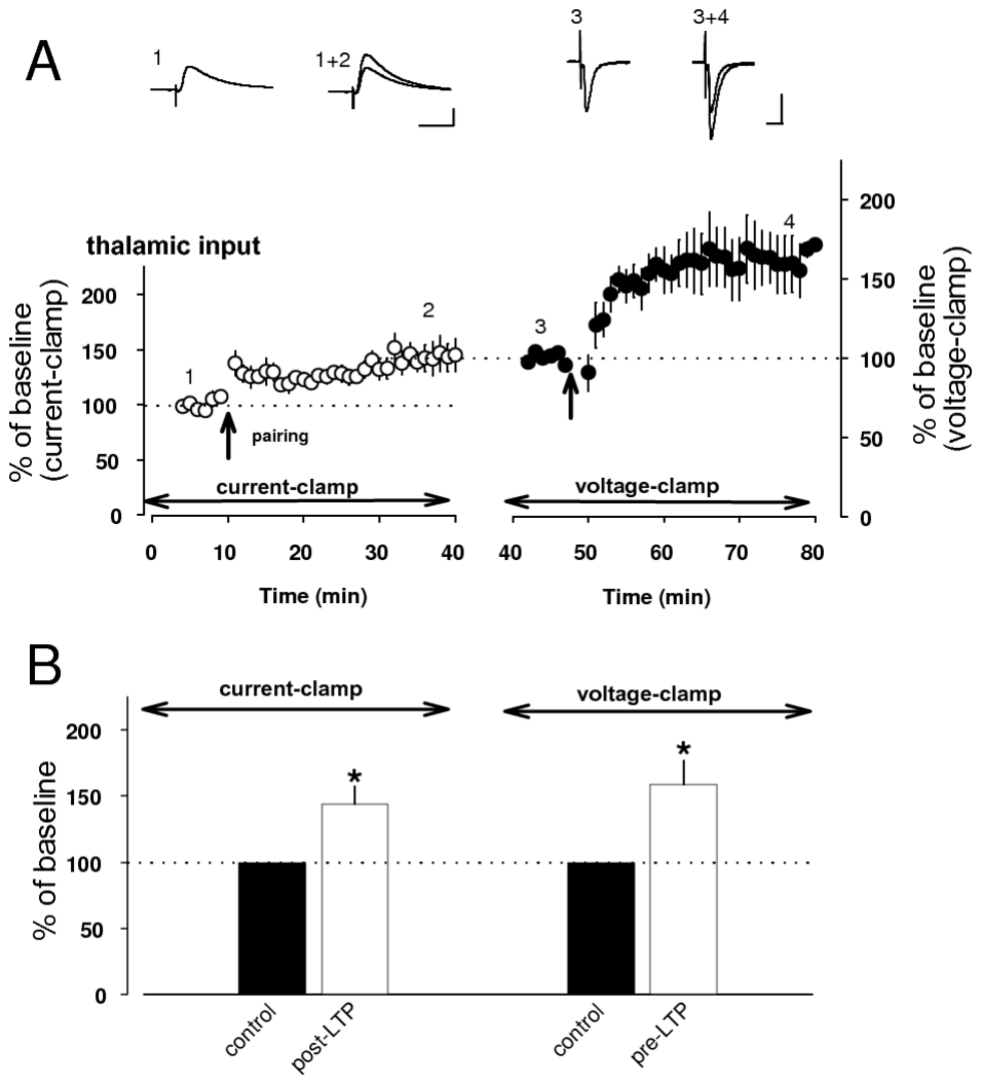
Post-LTP does not occlude the induction of pre-LTP. **A**, Post-LTP protocol, delivered to thalamic input, resulted in potentiation of EPSP to 144 ± 13% of baseline amplitude at thirty minutes after the induction (n=5, paired *t* test, p<0.05 versus baseline) in current–clamp mode. Recording mode was switched from current-clamp to voltage-clamp at thirty minutes after post-LTP induction. After EPSCs were evoked for the baseline, they were potentiated to 159 ± 17% of new baseline amplitude at thirty minutes by pre-LTP protocol (n=5, paired *t* test, p<0.05 versus baseline). Traces: averaged EPSP before (1) and after (2) post-LTP induction. Averaged EPSC before (1) and after (2) pre-LTP induction. Scale bar: 50 pA, 20 ms for voltage-clamp mode and 5 mV, 20 ms for current-clamp mode. **B**, Histogram showing quantification of results in **A**. * p<0.05.

Finally, we confirmed that the NMDAR-dependent form of LTP (post-LTP) did not occlude the induction of pre-LTP at the same thalamo-LA synapse (Figure 8A, B), leading to that these forms of synaptic plasticity are independent of each other.

Thus, these forms of synaptic plasticity may contribute to the encoding of conditioned fear memory by increasing independently the magnitude of synaptic response in thalamic input to the LA in different time scales.

**Figure 9:**
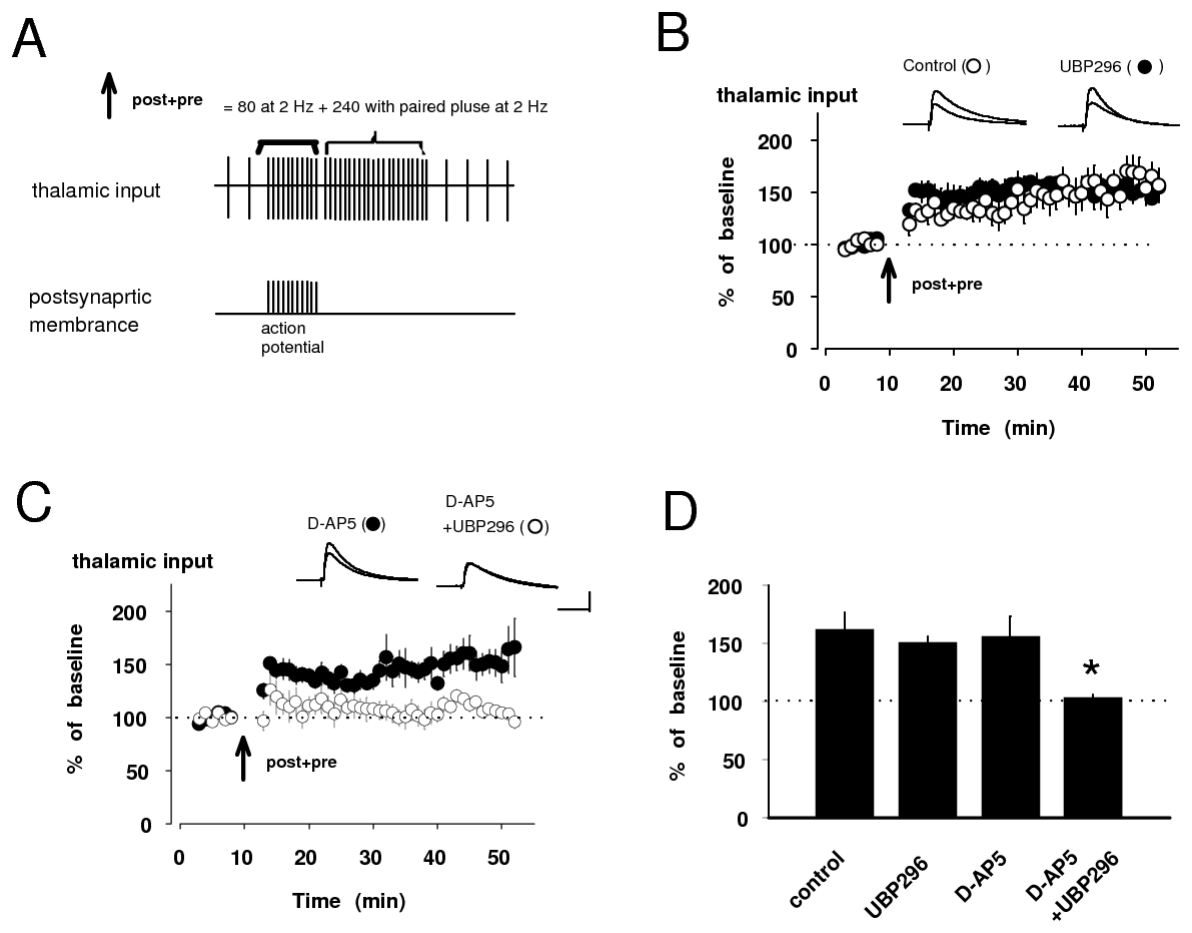
Precedent post-LTP protocol may prevent pre-LTP induction. **A**. Diagram illustrating combined protocols in which pre-LTP protocol follows post-LTP protocol with the delay of 0.5 s. **B**, **C**. The combined protocols, delivered to thalamic input, resulted in potentiation of EPSP to 162 ± 14% of baseline amplitude at thirty minutes after the induction (n=5, paired *t* test, p<0.05 versus baseline) in current–clamp mode (**B**). Neither UBP296 (**B**) nor D-AP5 (**C**) alone blocked this type of LTP. However, co-application of these antagonists markedly attenuated it (**C**). Scale bar: 5 mV, 50 ms. **D**. Histogram showing quantification of results in **B**, **C**. * p<0.05.

These inducibilities of post- and pre-LTPs, have been shown to be controlled by NO released from postsynaptic and presynaptic sites, respectively, based on the results of several experiments (Figures 4, 5, 6). We further presumed that endogenous NO produced by post-LTP mechanism might affect pre-LTP induction. To examine the crosstalk between pre- and post-LTP at the same thalamic input, we characterized LTP which was induced by post-LTP protocol and sequent pre-LTP protocol with the delay of 0.5 s under in current-clamp mode (Figure 9A). This combined protocol led to significant potentiation of EPSPs at the magnitude of 162 ± 14% of baseline value (n=5, paired *t* test, p<0.05 versus baseline, Figure 9B). We found that magnitude of LTP remained unchanged by either D-AP5 or UBP296 (UBP296: n=5, 150 ± 5% of baseline value, unpaired *t* test, p=0.45 versus control, D-AP5: n=6, 156 ± 17% of baseline value, unpaired *t* test, p=0.80 versus control, Figure 9B, C, D).

Interestingly LTP was not induced by the combined protocol under the presence of both D-AP5 and UBP296 (Figure 9C). The LTP induction (n=5, 103 ± 3% of baseline value) was significantly lesser than control (unpaired *t* test, p<0.01: Figure 9B, D) and treatment with UBP296 alone (unpaired *t* test, p<0.01: Figure 9B, D) or D-AP5 value (unpaired *t* test, p<0.05: Figure 9C, D), indicating that precedent post-LTP may prevent sequent pre-LTP. This result implicates that NO, released from postsynaptic site, may retrogradely prevent the induction of subsequent pre-LTP.

## Discussion

The present results indicate that repetitive low-frequency presynaptic activation in the absence of postsynaptic depolarization at thalamo-LA synapses leads to presynaptic KAR-dependent LTP (pre-LTP in thalamic input) [16]. Delivering of the same stimulation protocol to cortico-LA synapses does not lead to synaptic potentiation. However, EPSC in cortical input could be potentiated (pre-LTP in cortical input) when pre-LTP protocol is delivered in the presence of D-AP5. Both pre-LTPs are mechanistically similar, as they are completely blocked by selective antagonists of the GluR5 subunit-containing KAR, UBP296 and ACET [16] (Figure 2C). This result indicates that their induction process might require Ca^2+^ influx through calcium-permeable KAR in the LA. In this study, we could not assess the Ca^2+^-permeability of KAR in the LA, because this receptor was localized in presynaptic site (Figure 2A). However, a previous RT-PCR study documented that the fraction of the GluR5 mRNA in the adult rat amygdala contains an unedited form (approximately 30% of total mRNA), implying that the native GluR5 KAR in the LA is often Ca^2+^-permeable [22]. KAR composed of unedited subunits in glutamine/arginine site has the ability to permeate Ca^2+^, whereas KAR with edited subunits is Ca^2+^-impermeable [27]. These results implicate that KAR localized in cortical afferents in the LA may have the ability to permeate Ca^2+^.

Pre-LTP in both thalamic and cortical inputs are mechanistically different from either a previously described form of heterosynaptic plasticity that could be induced by activation of presynaptic NMDAR at cortico-amygdala synapse following paired stimulation of thalamic and cortical afferent fibers [28] or slowly developing form of heterosynaptic potentiation in the LA induced by prolonged low-frequency stimulation of cortical pathway alone [22]. Moreover, pre-LTP described here also differs from input timing-dependent plasticity, which is induced by paired thalamic and cortical stimulations in the amygdala [27].

It has been repeatedly demonstrated that long-term synaptic enhancement in either thalamic or cortical inputs to the LA constitute an essential mechanism contributing to encoding of fear memory [7,8,10,11,17,29,30]. Type of synaptic plasticity identified in this study is newly discovered plasticity forms that are observed in the LA when low-frequency stimulation is delivered to a single input alone without postsynaptic depolarization. These are also observed at physiological temperatures [16] (Figure 1L). The blockage of GluR5-containing KAR, which was involved in the formation of this plasticity [16] (Figure 2C), impaired fear memory [17], supporting a possibility that these plasticity mechanisms might contribute to encoding of conditioned fear. Our combined electrophysiological and behavioral study has revealed that fear conditioning occludes pre-LTP in thalamic input in slices prepared from conditioned rats. Interestingly, pre-LTP in cortical input was not occluded in the same conditioned rats. These results indicate that pre-LTP-like synaptic enhancements and consequent presynaptic KAR-dependent plasticity in thalamic, but not cortical, input to the LA might be recruited during fear conditioning, highlighting the importance of the direct thalamo-LA pathway in fear conditioning. Consistent with this result, auditory fear conditioning training was shown to induce substantial enhancements of the short-latency auditory responses, reflecting inputs from the auditory thalamus in freely moving rats [31] and fear conditioning was to potentiate synaptic efficacy at thalamo-LA synapses by a previous *in vivo* experiment [7]. A lesion study has revealed previously that activation of a single auditory input may be sufficient for fear conditioning [32]. Subsequent experiments demonstrated that LTP in both thalamic and cortical inputs, providing auditory conditioned stimulus information during conditioning, exhibit differential longevities and strength after conditioning in freely moving rats, suggesting distinct roles of these inputs in long-term memory [33,34].

The pre-LTP induction protocol utilized in this study is different from post-LTP protocols consisting of pairing of afferent stimulation with postsynaptic depolarization, fulfilling the well-known Hebbian rule on conventional NMDAR-dependent LTP. Its independence from somatic action potential firing differs strikingly from the Hebbian model, such as previously characterized spike-timing-dependent plasticity in the amygdala [18]. Principal neurons in the LA receive massive inhibitory inputs from the local GABA-releasing interneurons [35-37], which suppress the induction of spike timing-dependent LTP at thalamo-LA synapses [18,38-40]. Post-LTP protocol failed to induce LTP at thalamo-LA synapse with intact GABAergic inhibition [18], but weaker pre-LTP protocol could lead to robust synaptic potentiation despite strong GABAergic inhibition [18].

The patterns of neuronal activity in the LA of behaving animals during fear conditioning displayed increases in spontaneous activities [31], indicating that there is an increased number of enhanced postsynaptic depolarization of LA neurons during fear conditioning. A recent study demonstrated that pairing of an auditory cue with optically-induced action potentials in the LA pyramidal neurons (instead of a foot-shock) was sufficient for fear conditioning [41]. It has also been reported that norepinephrine or dopamine can gate LTP in thalamic input though suppression of feed-forward GABAergic circuit [38,40]. This is consistent with a report showing that responses reflecting the activity of thalamic areas are potentiated during fear conditioning [42]. In human studies, functional magnetic resonance imaging has also demonstrated that the amygdala shows activity changes during conditioning that correlate with activity in the thalamus [5]. Taken together, these results suggest that both membrane depolarization and blockade of GABAergic inhibition in response to aversive stimulus during conditioning training might promote conventional LTP. Consistently we demonstrated here that post-LTP was formed rapidly in the course of fear conditioning, whereas pre-LTP was induced more slowly after fear conditioning (Figure 7G, H). STM and LTM, parts of conditioned fear assessed at 1h and 24 h, respectively, after fear conditioning training were observed in conditioned animals [11,13]. Importantly infusion of drug disrupting NO signaling directly into the LA impaired LTM but not STM [13]. Consistent with this result, direct microdialysis showed that the percent of nitrate level, a marker of NO production, in the amygdala of conditioned animals was significantly higher than in the control at 80-150 min after fear conditioning, whereas the level of nitrate was hardly increased at 0-80 min [43]. These results suggest the possibility that pre-LTP may serve as main factors, whereas post-LTP does as an additional mechanism of synaptic strengthening in thalamic input to the LA contributing to encoding of fear memory.

In this study, we obtained evidence that pre-LTP in thalamic input could be blocked heterosynaptically by NO produced in response to activation of presynaptic NMDAR at cortico-LA synapses. However, the underlying cellular mechanism remains elusive. NO, produced in response to activation of postsynaptic NMDAR, has been previously known to serve as retrograde factor to promote post-LTP induction leading the increased probability of transmitter release in presynaptic site is well documented. Meanwhile, it has been already reported that NO produced by bath-applied NMDA before the induction, might suppress LTP in the hippocampus [23], leading to the possibility that NO has the ability to suppress synaptic plasticity via the increased threshold for LTP generation [44].

Many investigators have studied that conventional LTP, which could be induced by postsynaptic NMDAR activity, has been involved in encoding fear memory [13]. Our finding shows that the same type of LTP was blocked by a high concentration of Ca^2+^ chelator BAPTA in included in the recording pipette solution [18], implying that this form of LTP required postsynaptic Ca^2+^ influx for its induction (post-LTP). This potentiation was also prevented by either L-NAME, NOS inhibitor, or, PTIO, membrane-impermeable scavenger of NO (Figure 6E, F), indicating that NO may be produced in the postsynaptic site. However, this induction led to a substantial depression of paired pulse ratio of EPSPs (Figure 6C), suggesting that NO acts as retrograde factor and increases neurotransmitter release in the presynaptic terminal [45].

On the other hand pre-LTP was not blocked by BAPTA unlike post-LTP, but it was sensitive to pretreatment with BAPTA-AM, cell-permeable Ca^2+^ chelator (Figure 2A, D), suggesting that its induction requires presynaptic Ca^2+^ influx (pre-LTP). Although the mechanism underlying post-LTP are reasonably characterized, relatively little is known about molecular mechanism of pre-LTP. Nevertheless, previous studies suggested the some interesting possibilities that would be tested in future. Activation presynaptic N-type Ca^2+^ channel by 200-Hz stimulation at CA1 region of hippocampus may augment neurotransmitter release via the incorporation of additional N-type channels in the presynaptic membrane or an enhanced coupling between Ca^2+^ influx through the N-type Ca^2+^ channels and machinery of neurotransmitter release [46]. Importantly, it was shown that presynaptic form of LTP at cortico-LA synapse might be induced by direct activation of L-type Ca^2+^ channel [47]. Activation of KAR-induced Ca^2+^ influx and consequent enhanced membrane depolarization may therefore result in stimulation of both N-type and L-type Ca^2+^ channels.

We need to discuss the other mechanism for NMDAR-mediated blockage of pre-LTP because we have no direct result indicating that pre-LTP can produce NO in this study. The decreased synaptic strength, such as long-term depression (LTD), may decrease the magnitude of LTP. Presynaptic induced LTD has been reported to be induced by presynaptic NMDAR activation at layer4-layer 2/3 synapse, but, interestingly when postsynaptic NMDAR was activated, LTP was induced at the same synapse [47]. When these types of synaptic plasticity are evoked by same stimulation pattern, LTD might decrease the LTP value, resulting in the phenomenon that synaptic transmission remains unchanged.
